# Prevalence of *Borrelia* and *Rickettsia* in *Ixodes ricinus* from Chosen Urban and Protected Areas in Poland and the Czech Republic

**DOI:** 10.3390/insects15100785

**Published:** 2024-10-09

**Authors:** Dorota Kiewra, Dagmara Dyczko, Alena Žákovská, Helena Nejezchlebova

**Affiliations:** 1 Department of Microbial Ecology and Acaroentomology, University of Wrocław, 51-148 Wrocław, Poland; dagmara.dyczko2@uwr.edu.pl; 2 Institute of Experimental Biology, Faculty of Science, Masaryk University, 60200 Brno, Czech Republic; alenazak@sci.muni.cz (A.Ž.); helanej@sci.muni.cz (H.N.); 3 Department of Biology, Faculty of Education, Masaryk University, 60200 Brno, Czech Republic

**Keywords:** *Borrelia* spp., *Ixodes ricinus*, protected areas, *Rickettsia* spp., tick-borne risk, urban areas

## Abstract

**Simple Summary:**

*Ixodes ricinus*, the most important tick vector in Europe, is responsible for the spreading of medically important pathogens. This study aimed to detect *Borrelia* spp. and *Rickettsia* spp. in *I. ricinus* ticks collected in urban and protected areas both in Poland and the Czech Republic. *Ixodes ricinus* ticks collected in Poland compared to ticks from the Czech Republic were more infected with *Borrelia* spp., and ticks collected in protected areas were more infected with *Borrelia* spp. than ticks collected in urban areas. *Rickettsia* spp. prevalence in *I. ricinus* was comparable in both Poland and the Czech Republic; however, regional differences were observed. The regional differences in *Borrelia* spp. and *Rickettsia* spp. prevalence in *I. ricinus* indicate the need for adaptation of public health surveillance strategies in each region.

**Abstract:**

(1) Background: *Ixodes ricinus* is responsible for the spreading of medically important pathogens. Monitoring the level of tick infection in various areas is essential for determining the potential tick-born risk. This study aimed to detect *Borrelia* spp. and *Rickettsia* spp. in *I. ricinus* ticks collected in urban and protected areas both in Poland and the Czech Republic. (2) Methods: Ticks were collected by flagging in the years 2016–2017. *Borrelia* spp. was detected using nested PCR targeting the *flaB* gene and *Rickettsia* spp. using nested PCR targeting *gltA*. (3) Results: In total, DNA of *Borrelia* spp. was detected in 25.9% of samples. Ticks collected in Poland were more infected compared to the Czech Republic and ticks collected in protected areas were more infected with *Borrelia* spp. than ticks collected in urban areas. The RFLP analysis showed the occurrence of *B. afzelii* and *B. garinii* in both countries, and additionally *B. valaisiana*, *B. burgdorferi* s.s., and *B. miyamotoi* in Poland. *Rickettsia* spp. was detected in 17.4% of *I. ricinus*, with comparable infection level in both countries; however, regional differences were observed. (4) Conclusion: The regional differences in *Borrelia* spp. and *Rickettsia* spp. prevalence in *I. ricinus* indicate the complexity of factors influencing the level of infection and underline the need for adaptation public health surveillance strategies in each region.

## 1. Introduction

*Ixodes ricinus*, one of the best-known tick vectors in the world, is responsible for the spreading of tick-borne diseases in Europe, including Poland and the Czech Republic. Both in Poland and the Czech Republic, *I. ricinus* exists throughout the country [1,2,3] and is responsible for transmitting majority of tick-borne pathogens of medical importance, including Lyme borreliosis (LB). Lyme borreliosis remains still the most-often detected tick-borne diseases in Europe with the highest seroprevalence in countries in Western and Eastern Europe [4,5,6]. However, estimates of LB incidence vary not only in neighbouring countries, but also at the subnational level [5]. In Poland, over the last decade 2014–2023, according to the National Institute of Public Health NIH—National Research Institute [7], the incidence of LB ranged from 32.8 cases per 100,000 population in 2021 to 66.92 in 2023. In the Czech Republic, over the last decade 2014–2023, based on the data published by the National Institute of Public Health [8], cases of LB ranged from 25.7 cases per 100,000 population in 2021 to 42.9 cases in 2018. However, it is necessary to accept the idea that not all cases of the disease were reported to the system, so the actual number of cases may be higher. Furthermore, both in Poland and the Czech Republic, the variations in the number of notified cases are observed within the country. In Poland in 2022, when over 45.9/100,000 cases were recorded, the incidence ranged from 22.6 in Łódź voivodeship to 81.2 in Podlaskie voivodeship. In the Czech Republic, for example, in May 2023, during the spring period when high activity of ticks can be expected, 41 patients with LB were notified in the South Bohemian Region and only 3 patients in the capital city of Prague. The South Bohemian region is a rural part of the country where the population is approximately two-thirds that of Prague.

In addition to *Borrelia* spp., responsible for LB (*B. burgdorferi* s.l. group), *I. ricinus* can transmit *Borrelia*, responsible for relapsing fever (*B. miyamotoi* group), which are more recently described in human pathology [9]. *Borrelia miyamotoi*, first isolated from *I. persulcatus* ticks in Japan in the 1990s, is now known almost throughout the northern hemisphere, including Asia, Europe, and America. Infections caused by *B. miyamotoi* (*Borrelia miyamotoi* disease, BMD) are much less frequently diagnosed compared to *B. burgdorferi* s.l. [10]. The first case of *B. miyamotoi* spirochete infection in a human was noted in 2011 in Russia. Since then, *B. miyamotoi* infections have been diagnosed in North America, Asia (China, Japan), and Europe. In Poland, the first detection of *B. miyamotoi* DNA in a patient was described in 2019 [11].

*Ixodes ricinus* is also known to transmit tick-borne rickettsioses, including *Rickettsia helvetica*, which is frequently noted throughout Europe [12]. This *Rickettsia*, first isolated in 1979 from *I. ricinus* in Switzerland, was described in 1993 as a distinct species of the spotted fever group of rickettsiae named *R. helvetica* [13]. It seems that the pathogenicity of *R. helvetica* varies between patients, from asymptomatic infection with non-specific clinical signs with mild and self-limiting courses to serious symptoms [9,12,14,15]. In Poland, according to the National Institute of Public Health NIH—National Research Institute [7], a few human cases of spotted fever and other rickettsioses are noticed per year; however, it is not distinguished whether they are caused by *R. helvetica* or another *Rickettsia* species. In the Czech Republic, rickettsioses and the occurrence of antibodies against *Rickettsia* spp. are not routinely investigated. Although it has been confirmed that ticks in the Czech Republic contain *Rickettsia* spp., only rare cases of human infections were reported. On the website of the State Health Institute [8], the number of confirmed cases of rickettsiosis can be found, showing that in past 10 years, a total of 58 cases have been reported. However, it is not possible to trace a specific type of disease caused by a certain type of pathogenic rickettsia. Moreover, rickettsioses do not show any characteristic symptoms such as fever and rash, but even then they can easily be confused with other diseases with similar symptoms. *Rickettsia slovaca*, transmitted mainly by ticks of the *Dermacentor* genus and *R. monacensis* and *R. helvetica*, which are transmitted by the *I. ricinus*, are considered to be the main candidates for spreading this type of infection in the Czech Republic [8].

The increase in outdoor activities among Europeans extends contact with ticks and consequently increases the risk of tick-borne diseases [16]. A rise in the incidence of LB over the last decades and the detection of new tick-borne pathogens indicates the need for systematic monitoring the potential health tick-borne risks. In Central Europe, *I. ricinus* inhabits mainly broad-leaved and mixed forests and forest-like habitats, but the tick can also be found in suburban locations [17]. Moreover, infected *I. ricinus* may be present both in forest and urban green spaces, including urban parks and gardens with suitable microclimate and host availability [18]. Therefore, determining the level of tick infection in various areas, including diversity in geographical location and the impact of anthropopressure, is crucial for determining potential tick-born risk. This study aimed to assess the infection level with *Borrelia* and *Rickettsia* among *I. ricinus* ticks collected in chosen urban and protected areas both in Poland and the Czech Republic.

## 2. Materials and Methods

### 2.1. Tick Sampling

**Figure 1:**
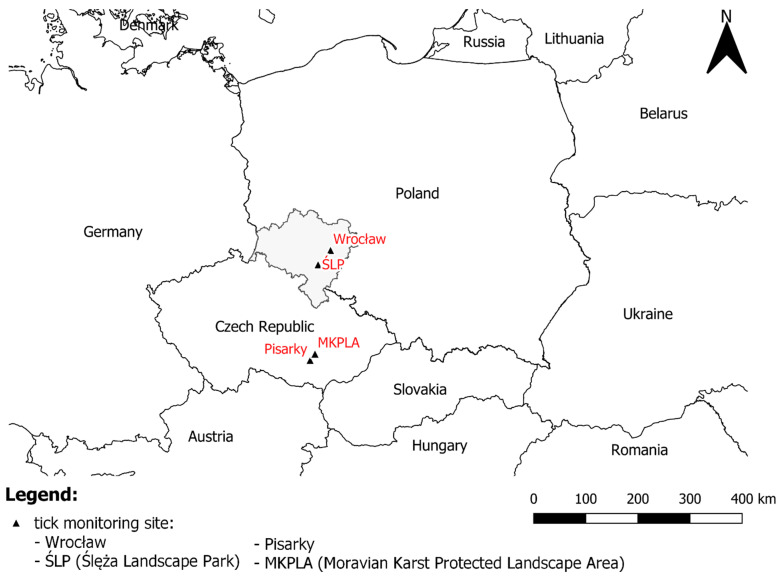
Tick monitoring sites in Poland and the Czech Republic.

Ticks were collected by flagging vegetation in the years 2016–2017 in four localities (Figure 1), including urban and protected areas both in south-western Poland and south-eastern Czech Republic. Urban areas covered city parks in communal forests in the conurbation of Wrocław (Poland) and Brno (the Czech Republic), while protected areas were located in forests of the Ślęża Landscape Park (Poland), and the Moravian Karst Protected Landscape Area (the Czech Republic). Wrocław (51°6′ N 17°2′ E), the capital of the Lower Silesia region located on the banks of the Odra River, 105–156 m above sea level, is the fourth largest city in Poland with a population of ca 640,000 inhabitants. The town is characterised by a temperate transitional climate subject to continental and oceanic influences, with the annual average temperature estimated at ca 10 °C and a mean annual precipitation of 567 mm [19]. The urban green spaces cover 34.0% of the city area and a total area of parks and communal forests in Wroclaw account for ca 530 ha [19]. The Ślęża Landscape Park (ŚLP), established in 1988, with the highest elevation ofŚlęża (717 m a.s.l.), is located about 40 km southwest of Wrocław and covers an area of ca 7600 ha [20]. The ŚLP is very popular among tourists and cyclists because the natural habitats in ŚLP include forest, rocky, meadow, and grassland habitats threatened due to anthropogenic impacts [21]. To protect the most valuable floristic, landscape, and historical areas, three nature reserves were created within ŚPK. In Brno (49°12′ N 16°37′ E), the conurbation of the locality of Pisarky was chosen. Pisarky, located 2 km far from the centre of the town, is situated 197–210 m above sea level and forms the bottom of Pisarky vale. On the hillside of this valley, a mixed wood as a park wood for the people’s holiday utilisation is growing. In this biotype, the predominant trees are *Quercus petraea*, *Q. robur*, *Carpinus betulus*, and *Pinus silvestris*. The annual average temperature is ca 8.9 °C and the mean annual precipitation is 559 mm. The environment of the Moravian Karst Protected Landscape Area (MKPLA, 49°21′ N, 16°42′ E) is situated in the surrounding of Skalní mlýn, with preserved species in beech forests. In the upper warmer parts, there are oak and horn beam forests with rivers and extremely wet meadows. The site extends around the emergence of the river Punkva, the best-developed karst phenomena. The annual average temperature is estimated at ca. 7.7 °C and a mean annual precipitation of 600 mm, situated 334 m above sea level. The collected ticks were identified at the species level with the use of a key for Ixodida identification [22].

#### *Borrelia* spp. and *Rickettsia* spp. Detection

The molecular study covered 413 individual *Ixodes ricinus* ticks, including 235 ticks (190 nymphs, 27 females, 18 males) collected in Poland (91 in Wrocław and 144 in ŚLP), and 178 ticks (119 nymphs, 30 females, 29 males) from the Czech Republic (72 from Brno and 106 from MKPLA). Both nymphs and adults were tested individually, and DNA extraction was carried out with the ammonium hydroxide (NH_4_OH) method by Stańczak et al. [23]. For the detection of *Borrelia* spp., the specific nested PCR targeting *flaB* gene-encoding flagellin [24], and for the detection of *Rickettsia* spp., primers targeting *gltA* gene-encoding citrate synthase were used [25,26]. Both nested PCR reactions were carried out with the use of PCR Mix Plus (A&A Biotechnology, Warszawa, Poland) and Thermal Cycler T100 (BioRad T100™ Thermal Cycler, Warszawa, Poland). The PCR mixture for *Borrelia* (25 μL total volume) consisted of 12.5 μL 2 × PCR Mix Plus (A&A Biotechnology), 2.5 μL of each primer, 4.5 μL sterile nuclease-free water, and 3 μL of template DNA for the first reaction, and 12.5 μL-2 × PCR Mix Plus (A&A Biotechnology), 2.5 μL of each primer, 5.5 μL sterile nuclease-free water, and 2 μL of the outer PCR product for nested PCR. The *flaB* gene for *Borrelia* spp. was amplified using the following thermal cycling programme: 95 °C for 3 min, 35 cycles at 95 °C for 45 s, 50 °C (132f i 905r) or 54 °C (220f i 824r) for 45 s, 72 °C for 1 min, followed by a final extension at 72 °C for 5 min. The reaction mixture for a single sample for *Rickettsia* spp. had a volume of 25 μL: 12.5 μL 2 × PCR Mix Plus (A&A Biotechnology), 1.25 μL of each primer, 5 μL sterile nuclease-free water, and 5 μL of template DNA for the first reaction, and 12.5 μL-2 × PCR Mix Plus (A&A Biotechnology), 1.25 μL of each primer, 8 μL-sterile nuclease-free water, and 2 μL of the outer PCR product for nested PCR. PCR reactions for *Rickettsia* spp. were as follows: 95 °C for 2 min, 35 cycles at 95 °C for 30 s, 50 °C (877p and 1258n) or 48 °C (896p and 1233n) for 30 s, and 72 °C for 1 min at the final elongation step at 72 °C for 5 min.

The PCR products were separated on 1.5% agarose gel (30 min, 100 V), and results were observed under a UV light transluminator. For identifying the genospecies of *Borrelia* spp., the RFLP method was used. The nested PCR positive samples were treated with the restriction enzyme HpyF3I (FastDigest HpyF3I, ThermoScientific, Waltham, MA, USA) according to Wodecka [27]. Restriction patterns obtained after electrophoresis in 3% agarose gel (75 min, 65 V) were observed under UV light and assigned to appropriate genospecies.

### 2.2. Statistic

To assess the relationship between pathogen infection levels and tick developmental stages (nymphs, females, and males), as well as between pathogen infection levels and study areas, a Chi-square test with *p* < 0.05 was used. Statistical analyses were performed using Statistica software version 12.0.

## 3. Results

In total, the DNA of *Borrelia* spp. was detected in 25.9% (107/413) of ticks (Table 1), with males being more infected (44.7%) compared to nymphs (23.3%) and females (24.5%) (χ^2^ = 9.777, *p* = 0.0075). Infected ticks were found both in the Czech Republic and Poland; however, *I. ricinus* collected in Poland were more infected compared to the Czech Republic (36.2% and 12.9%, respectively; χ^2^ = 28.348, *p* < 0.001). *Ixodes ricinus* collected in protected areas both in Poland and the Czech Republic were more infected, with *Borrelia* spp. than ticks collected in urban areas (48.6% vs. 16.5% in Poland, and 18.9% vs. 4.2% in the Czech Republic; χ^2^ = 24.93, *p* < 0.001; χ^2^ = 8.236, *p* = 0.0041, respectively).

The RFLP analysis of 57 samples positive in nested PCR proved five restriction patterns: four from the *B. burgdorferi* s.l. complex (*B. afzelii*, *B. garinii*, *B. valaisiana*, and *B. burgdorferi* s.s.) and one *B. miyamotoi* (Table 2). The dominant species of *Borrelia* spp. was *B. garinii* (42%), followed by *B. afzelii* (35%). *Borrelia valaisiana* (9%) and *B. burgdorferi* s.s. (3.5%) were found less frequently. *Borrelia afzelii* and *B. garinii* were detected both in Poland and the Czech Republic, whereas *B. valaisiana* and *B. burgdorferi* s.s. were only detected in Poland. *Borrelia miyamotoi* was identified in 10.5% *Borrelia* samples. Moreover, *B. miyamotoi* was found only in Poland, both in urbanised and protected areas.

*Rickettsia* spp. was detected in 17.4% (72/413) of *I. ricinus* (Table 1). Females were more infected compared to nymphs and males (33.3% vs. 14.6% and 17.0%; χ^2^ = 12.032, *p* = 0.002). The overall level of infection recorded in Poland and the Czech Republic was comparable, and the share was 18.7% and 15.7%, respectively (χ^2^ = 0.006, *p* = 0.9383). However, in the Czech Republic, more infected ticks were found in MKLPA than in Brno (21.7% vs. 6.9%; χ^2^ = 7.041, *p* = 0.008) and in Poland more in Wrocław than in ŚLP (26.4% vs. 13.9%; χ^2^ = 5.711, *p* = 0.0169).

Of the total 413 ticks, 13 ticks (3.0%), including 3 from MKLPA, 5 from ŚPK, and 5 from Wrocław, were co-infected with *Borrelia* spp. and *Rickettsia* spp. (Table 3). Co-infections were most often found in females and were less frequently found in males and nymphs (χ^2^ = 7.577, *p* = 0.0226). Additionally, in Wrocław, also, one co-infection of *B. garinii* and *B. miyamotoi* was noted.

## 4. Discussion

In the present study, the DNA of *Borrelia* was detected in *Ixodes ricinus* collected both in the Czech Republic and Poland; however, one of the most striking observations from our study is the significantly higher prevalence of *Borrelia* spp. among tick populations in Poland compared to the Czech Republic (36.2% vs. 12.9%). A potentially higher level of tick infection with *Borrelia* in Poland than in the Czech Republic has also been shown in other studies. The extensive literature review on *B. burgdorferi* s.l.’s prevalence in *I. ricinus* in peri/urban areas across Europe during the period 1990–2021 showed that adults and nymphs collected in Poland seem to be more infected than collected in the Czech Republic [18]. This finding suggests a notable difference in the risk of Lyme borreliosis (LB) transmission between the two countries. Several factors may contribute to this discrepancy, including the composition of tick microbiome and interactions within it, the viability of observed pathogens and their sensitivity to different factors, the variations in habitat characteristics, climatic conditions, the abundance and diversity of host species populations, the abundance and activity of ticks and vector species in general, landscape management, and many other factors which can influence pathogen transmission dynamics [28,29,30]. The difference in *Borrelia* spp.’s prevalence in Poland compared to the Czech Republic observed in our study underscores the need for tailored public health interventions and surveillance strategies in each region. In Poland, where *Borrelia* prevalence is notably higher, targeted efforts to raise awareness about LB risk and promote preventive measures such as tick avoidance and prompt tick removal are imperative. The need to raise awareness of tick-borne diseases was also found in surveys carried out among Czech and Polish university students of science [31]. The results obtained from the questionnaires show that the students of both countries were aware of the tick-born risks; however, they had only a superficial knowledge concerning tick-borne infections. Furthermore, the Czechs felt more at risk by ticks, and compared to the Poles, recognised the repellents as more effective.

In both countries, *I. ricinus* ticks collected in protected areas were more infected with *Borrelia* than ticks collected in urban areas. A similar pattern was noted in Slovakia, where the lowest prevalence was detected in the urban/suburban habitat than in the natural and agricultural habitat [32]. However, the result is not consistent with the meta-analysis of *B. burgdorferi* s.l.’s prevalence in questing *I. ricinus* in Europe, which shows that prevalence in peri/urban green space appears to be slightly higher compared to the average prevalence in Europe, which includes both urban and rural locations [18]. Nevertheless, this finding suggests that urbanisation through changes in host composition and modifications of habitat are likely to have an impact on *Borrelia* infection levels in ticks. However, a thorough understanding of the impact of urbanisation should be further investigated. Additionally, enhanced surveillance of tick populations in a diverse range of environments, understanding the effects of local habitat, and thorough analyses of LB human cases can help identify high-risk areas and guide targeted interventions.

In Europe, the species from the *B. burgdorferi* s.l. group most often causing LB in humans include *B. burgdorferi* s.s., *B. garinii*, *B. afzelii*, and, less frequently, *B. spielmanii* and *B. bavariensis* [9]. In our study, we found that, in both countries, *B. garinii* and *B. afzelii* were the most often detected genospecies. It is in line with a previous study conducted in Europe, where the meta-analysis of reports published between 2010 and 2016 shows that *B. afzelii* and *B. garinii* were the most frequently detected genospecies [33]. The presence of both *B. afzelii* and *B. garinii* indicates a diverse animal reservoir because *B. afzelii* is usually linked to rodents and *B. garinii* to birds [9]. Additionally, in Poland, in the *B. burgdorferi* s.l. complex, *B. valaisiana* and *B. burgdorferi* s.s. were determined, but they occurred less frequently. The knowledge of the distribution of particular genospecies is particularly important for epidemiologists due to various clinical outcomes [34].

In our study, among the identified *Borrelia* species, in the Czech Republic only spirochetes from the *Borrelia burgdorferi* s.l. group were found, while in Poland, in addition to *B. burgdorferi* s.l., *B. miyamotoi* was also noted. Perhaps the lack of *B. miyamotoi* presence in the Czech Republic is due to the relatively small number of ticks tested, because other studies show the presence of *B. miyamotoi* in the Czech Republic tick population. However, the estimated prevalence is low. Crowder et al. [35] detected *B. miyamotoi* in 8 ticks among 435 tested (1.8%), Honig et al. [36] in 2.1% (in 9 of the 435), and Bubanová et al. [37] in 2.0% (in 10 of the 505). In Poland, the presence of *B. miyamotoi* was confirmed in questing *I. ricinus* both in urban and wild areas; however, the prevalence was lower than in the case of LB spirochetes [38,39].

In contrast to *Borrelia*, our analysis reveals a comparable prevalence of *Rickettsia* spp. between Poland and the Czech Republic; however, differences in prevalence were observed at the subnational level. In the Czech Republic, more infected ticks were found in landscape parks than in urban areas (21.7% vs. 6.9%); in Poland, on the contrary, the infection was higher in urban areas than in landscape park (26.4% vs. 13.9%). The long-term study at Pisárky Park, i.e., locality covered also by current research, showed that from a total of 2813 *I. ricinus* ticks collected from 1996 to 2002, the mean positivity was 5.8% [40], which is similar to our results at the same place. A previous study conducted in the Czech Republic showed a higher minimal infection rate (3.4%) in a natural rather than an urban (2.9%) ecosystem [41], whereas in Poland, the prevalence of *Rickettsia* spp. was higher in urban (6.5%) than natural (4.4%) areas [42]. However, it is worth emphasising that information on the occurrence of *Rickettsia* species in ticks both in the Czech Republic and Poland is still fragmentary. The variability in *Rickettsia* spp.’s prevalence underscores the complex epidemiological dynamics of tick-borne pathogens and highlights the importance of continuous disease risk assessments and monitoring. The congruence in *Rickettsia* spp.’s prevalence suggests that certain ecological and environmental factors may exert a uniform influence on the transmission of this pathogen across borders. In both Poland and the Czech Republic, the prevalence of *Rickettsia* spp. determined in our study highlights the importance of vigilance linked with tick-borne pathogens beyond the causative agent of Lyme borreliosis. Exposure to tick-borne *Rickettsia* is also evidenced by serological tests of foresters, which are a group particularly exposed to tick bites [14]. Given the potential medical importance of rickettsial infections, proactive measures to reduce tick exposure and improve diagnostic capabilities are essential.

From a medical point of view, co-infections may be of particular importance due to its possible impact on severity of the disease in humans. In our studies, we found co-infection *Rickettsia* and *Borrelia* spp. in 3% of *I. ricinus*, with females being more frequently co-infected compared to nymphs and male ticks. Although we did not confirm interactions between *Borrelia* and *Rickettsia* infection due to the small sample size, such an association was shown in a meta-analysis conducted by Raulf et al. [43]. Therefore, the coexistence of different pathogens should be considered to be one of the factors influencing the level of prevalence.

## 5. Conclusions

Our study points out the regional differences in *Borrelia* spp. and *Rickettsia* spp. prevalence between Poland and the Czech Republic. By identifying these differences, we provide new insights for public health authorities and policymakers to develop targeted strategies for tick-borne disease prevention and control. However, further research is needed to clarify the underlying factors driving these discrepancies and to enhance our understanding of tick-borne pathogens issues in diverse ecological habitats.

## Figures and Tables

**Table 1:** *Borrelia* spp. and *Rickettsia* spp. detected in *Ixodes ricinus* ticks collected in urban and protected areas in Poland and the Czech Republic.

County	Site	*Ixodes ricinus* Developmental Stage Number of Infected/Number of Investigated							
		*Borrelia* spp.				*Rickettsia* spp.			
		Nymph	Female	Male	Total	Nymph	Female	Male	Total
Czech Republic	Brno	3/61	0/5	0/6	3/72 (4.2%)	3/61	1/5	1/6	5/72 (6.9%)
	MKLPA	12/58	0/25	8/23	20/106 (18.9%)	7/58	11/25	5/23	23/106 (21.7%)
	Total	15/119	0/30	8/29	23/178 (12.9%)	10/119	12/30	6/29	28/178 (15.7%)
Poland	Wrocław	11/69	4/17	0/5	15/91 (16.5%)	17/69	7/17	0/5	24/91 (26.45)
	ŚLP	47/121	10/10	13/13	70/144 (48.6%)	18/121	0/10	2/13	20/144 (13.9%)
	Total	58/190	14/27	13/18	85/235 (36.2%)	35/190	7/27	2/18	44/235 (18.7%)
Total		72/309 (23.3%)	14/57 (24.5%)	21/47 (44.7%)	107/413 (25.9%)	45/309 (14.6%)	19/57 (33.3%)	8/47 (17.0%)	72/413 (17.4%)

**Table 2:** *Borrelia* genospecies detected in *Ixodes ricinus* ticks collected in Poland and the Czech Republic.

County	*Ixodes ricinus*	*Borrelia burgdorferi* s.l. (%)				*B. miyamotoi* (%)
		*B. afzelii*	*B. garinii*	*B. valaisiana*	*B. burgdorferi* s.s.	
Czech Republic	Nymph	3	1	-	-	-
	Female	-	-	-	-	-
	Male	1	-	-	-	-
	Total	4	1	-	-	-
Poland	Nymph	12	9	3	1	5
	Female	2	10	2	1	-
	Male	2	4	-	-	1
	Total	16	23	5	2	6
Total		20 (35%)	24 (42%)	5 (9%)	2 (3.5%)	6 (10.5%)

**Table 3:** Coinfection of *Borrelia* spp. and *Rickettsia* spp. in *Ixodes ricinus* ticks collected in Poland and the Czech Republic.

County	Site	*Ixodes ricinus* Developmental Stage Number of Coinfected/Number of Investigated (%)			
		Nymph	Female	Male	Total
Czech Republic	Brno	0/61	0/5	0/6	0/72
	MKLPA	1/58	2/25	0/23	3/106
Poland	Wrocław	2/69	3/17	0/5	5/91
	ŚLP	3/121	0/10	2/13	5/144
Total		6/309 (1.9%)	5/57 (8.8%)	2/47(4.3%)	13/413(3.0%)

## Data Availability

All data are contained within the manuscript.
